# Oral health and rehabilitation in face transplant recipients – a systematic review

**DOI:** 10.1007/s00784-024-06078-3

**Published:** 2025-01-06

**Authors:** Leonard Knoedler, Martin Kauke-Navarro, Samuel Knoedler, Tobias Niederegger, Elena Hofmann, Max Heiland, Steffen Koerdt, Susanne Nahles, Helena Baecher

**Affiliations:** 1 Department of Oral and Maxillofacial Surgery, Charité – Universitätsmedizin Berlin, corporate member of Freie Universität Berlin, Humboldt- Universität zu Berlin, Berlin Institute of Health, Berlin, Germany; 2 Department of Surgery, Division of Plastic Surgery, Yale School of Medicine, New Haven, CT USA; 3 Department of Cranio- and Maxillofacial Surgery, University Hospital Regensburg, Regensburg, Germany; 4 Berlin Institute of Health (BIH) Charité Junior Clinician Scientist Program, Berlin Institute of Health at Charité - Universitätsmedizin Berlin, Berlin Institute of Health Biomedical Innovation Academy, Charitéplatz 1, 10117 Berlin, Germany

**Keywords:** Face transplantation, Vascularized composite allotransplantation, VCA, Oral health, Oral rehabilitation

## Abstract

**Background:**

Facial transplantation (FT) provides advanced solutions for severe facial defects by incorporating complex tissues such as bone, skin, oral mucosa and nerves. Oral health plays a critical role in FT, impacting both functional outcomes and transplant prognosis. Despite its importance, literature on oral health in FT recipients remains sparse.

**Methods:**

This systematic review adhered to PRISMA guidelines. We searched PubMed, EMBASE, Web of Science, and CENTRAL for studies on oral health in FT patients up to August 4, 2024. Eligible studies were evaluated using the Newcastle-Ottawa Scale (NOS) and Level of Evidence (LOE). Data extracted included study design, general information on FT, patient demographics, oral examination techniques, and outcomes.

**Results:**

Out of 6,984 articles reviewed, 19 met the inclusion criteria, all consisting of case reports or case series involving 48 FT cases. Of these, 25 cases provided relevant oral health and rehabilitation data. All studies showed a LOE of IV, with an average NOS score of 4.3 **±** 0.5. Most FT cases involved male recipients (*n* = 20, 80%), while the majority of FTs were conducted in the United States (*n* = 10, 40%) and France (*n* = 7, 28%). Oral mucosa biopsy was the most common examination method (*n* = 11, 44%). Malocclusion was reported in 48% (*n* = 12) of cases, with revision surgeries occurring in 36% (*n* = 9). Post-FT dental treatments included tooth extractions (*n* = 7, 28%), fillings (*n* = 3, 12%), and endodontic treatments (*n* = 2, 8%). Dental implants were placed in 32% (*n* = 8) of cases, with one case (4%) reporting failed osseointegration.

**Conclusion:**

Routine oral health assessments are essential for FT patients to prevent complications and improve clinical outcomes. However, there is a lack of high-quality research on oral health in FT recipients, underscoring the need for further studies to establish standardized care protocols.

**Clinical relevance:**

This review emphasizes the urgent need for standardized oral health protocols in FT patients to minimize infection risks and optimize long-term transplant success and overall patient health.

**Supplementary Information:**

The online version contains supplementary material available at 10.1007/s00784-024-06078-3.

## Introduction

Facial transplantation (FT) represents a groundbreaking biotechnology that aims to restore form and function [1]. Comprised of various tissue types, FT can include bone, skin, muscle, oral mucosa, cartilage, blood vessels, and nerves [2]. Over the past two decades, FT has shown promising outcomes in patients with irreversible tissue loss and severe facial defects. FT not only expands the reconstructive ladder but also helps reduce the psychological burden and social isolation following extensive facial trauma [3]. The potential to improve quality of life, including significant psychological aspects, must be carefully weighed against the drawbacks of lifelong immunosuppression. To date, at least 48 full and partial FTs have been performed worldwide, of which at least 30 included maxillary or mandibular bone transplants [1]. 

Oral health is known to be a significant health factor that impacts patient longevity and perioperative risk profile [4]. Vice versa, a wide array of clinical factors such as the nutritional status, smoking, or obesity, has been shown to influence oral health. Recent literature has suggested that optimal oral health represents a promising therapeutic target to optimize patient outcomes [5]. 

In cases where FT involves not only the facial skin but also additional structures such as portions of the maxilla and mandible, oral mucosa, temporomandibular joint, tongue, floor of the mouth, and teeth, the functional considerations—such as mastication, swallowing, speech, and oral competence—become even more complex. As a result, oral rehabilitation in these scenarios is more intricate and poses significant challenges for the entire transplantation team. The aims of post-transplant dentition include: (i) preserving the recipient’s own teeth, (ii) preserving the transplanted teeth, and/or (iii) improving dental status by implementing dental implants and prosthetics [6]. 

Several reviews on FT have been published, reporting on both short- and long-term outcomes, including patient and graft survival, functional outcomes (e.g., breathing, eating, speech, and facial expression), as well as surgical revisions following FT [7]. However, there is a notable lack of up-to-date review literature specifically addressing oral health and oral rehabilitation in FT recipients. Previous work has mainly been focused on isolated aspects of oral health in FT patients [8]. Thus, future research is warranted to review and condense our knowledge on oral health in FT recipients.

In this systematic review, we aim to summarize the current body of evidence, as well as the challenges and controversies in this field. Ultimately, this line of research may highlight future research directions and unlock translational potential to improve outcomes in FT recipients.

## Methods

This systematic review adhered to the Preferred Reporting Items for Systematic Reviews and Meta-Analyses (PRISMA) 2020 guidelines. A narrative synthesis was performed as a meta-analysis was deemed unsuitable due to the heterogeneity in outcome measures.

### Systematic search

A comprehensive review was conducted by screening the PubMed/MEDLINE, Web of Science, EMBASE, and CENTRAL databases up to August 4, 2024. The search strategy comprised three elements combined using “AND”. The search terms included: i) “facial” OR “face” AND ii) “transplant” OR “VCA” OR “vascularized composite allograft” OR “vascularized composite allotransplantation” OR “allograft” OR “implant” AND iii) “oral health” OR “oral rehabilitation” OR “oral care” OR “intraoral” OR “dental implants” OR “prosthesis”.

The chosen articles were required to be authored in English and accessible as full-text. Exclusion criteria included animal studies, cadaver studies, and non-surgical investigations. Titles and abstracts were independently screened by two reviewers (H.B. and T.N.), followed by a detailed manual examination of all eligible full-text articles. When multiple publications pertained to the same patient cohort, the study providing the most comprehensive information on oral health and featuring the longest follow-up period was selected. In case of any discrepancy, a third reviewer (L.K.) was consulted.

**Fig. 1:**
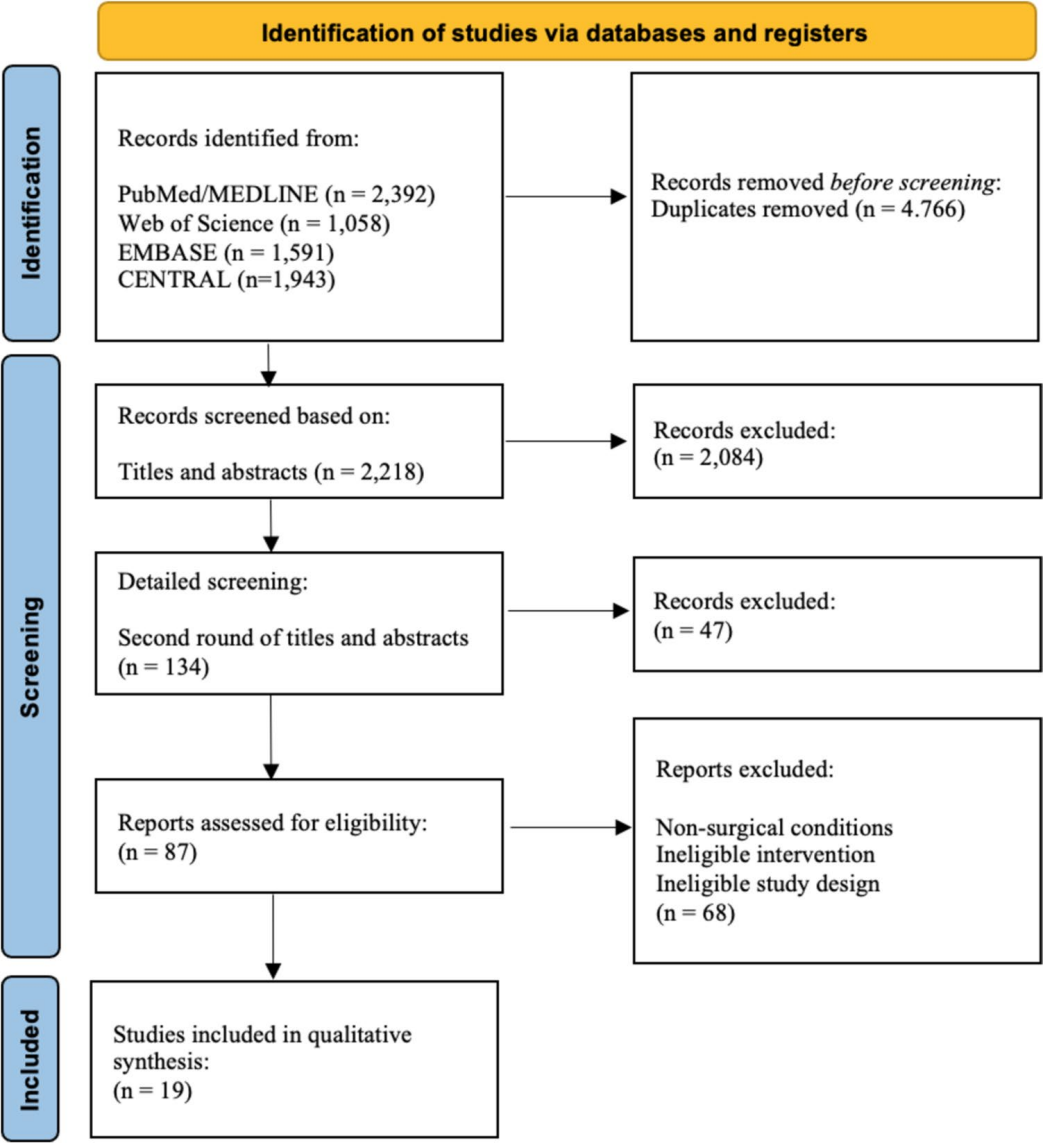
PRISMA 2020 flow diagram of the study identification process

The search strategies used for each database are detailed in Supplementary Digital Content 1. The PRISMA 2020 flowchart, shown in Fig. 1, outlines the selection process and inclusion/exclusion criteria.

### Quality assessments

To assess the quality of the selected papers, we employed the Newcastle–Ottawa Scale (NOS) and the Level of Evidence (LOE). The NOS assigns up to nine stars to each study, evaluating three key domains: selection of study cohorts (maximum of four stars), comparability of groups (maximum of two stars), and assessment of outcomes/exposures (maximum of three stars). A higher NOS score indicates superior study quality and a reduced risk of bias [9]. The LOE provides a hierarchical ranking of various study types based on their methodological rigor and susceptibility to bias. LOE I represents studies with high methodological quality, such as systematic reviews or meta-analyses derived from well-conducted randomized controlled trials [10]. 

### Data extraction

Using a blinded, dual-review process, we extracted the following variables for each article included in this review: Digital Object Identifier (DOI), first author, year of transplantation, study design, sample size, recipient age and gender, donor age and gender, immunosuppression, indication for FT, length of follow-up, postoperative dentition, postoperative dental treatments, occlusion, postoperative oral examinations, dental rehabilitation, functional outcome, bone infection, mucosal condition, salivary gland function, lip competence, oral sensation, patient satisfaction, and revision rate. The collected data were stored in a Microsoft Excel file and analyzed using GraphPad Prism (v9.00 for macOS, GraphPad Software, La Jolla, CA, USA), which was also used for data visualization.

## Results

### General study characteristics

A comprehensive literature search identified 6,984 articles, of which 19 met the predetermined inclusion and exclusion criteria.

All included studies comprised case reports or case series. Given the limited number of FT cases performed worldwide, the studies were organized by individual FT cases. A total of 48 FT cases were initially identified across all investigated studies (Supplementary Digital Content 2). Of those, 25 cases providing information on oral health and rehabilitation were included in this study.

Of all studies reviewed, in 19 articles (100%) the level of evidence (LOE) was classified as IV. The average Newcastle-Ottawa Scale (NOS) score was 4.3 **±** 0.5. Detailed evaluations of the NOS and LOE are provided in Supplementary Digital Content 3 and 4.

Notably, five cases (20%) of FT were identified in female patients and 20 cases (80%) in male patients. The recipient age ranged from 19 to 64 years, while the donor age ranged from 21 to 60 years. Among the selected studies, ten (40%) FTs were performed in the United States, while seven (28%) were conducted in France. Additionally, two (8%) FTs were each performed in Spain, Turkey, and Finland, while one FT (4%) was performed in Belgium and Canada, respectively. Overall, four (16%) cases of the FTs involved exclusively soft-tissue-only vascularized composite allotransplantation (VCA), while 21 (84%) cases included the transplantation of skeletal components of the maxilla or mandible. In 17 (68%) of these transplantations, at least one tooth was transplanted. The mortality rate was 28%, with seven out of 25 FT recipients deceased at the time of data extraction. General data and information on oral health of the included FT cases are provided in Tables 1 and 2.

**Table 1:** General data of all included studies ordered by FT cases

Year of FT	Patient	Reason for facial defect	Country of transplantation	Recipient age, sex (f: female, m: male)	Donor age, sex (f: female, m: male)		Reconstructed area	Status
2005	1 [11]	Animal attack	France	38, f	46, f	Nose, lips, chin, cheeks		Died
2007	2 [12]	Neurofibromatosis	France	29, m	N/A	Facial soft tissue		Alive
2008	3 [13]	Ballistic trauma	US	45, f	Age matching, f	Midface soft tissue, nose, cheeks, upper lip, right globe, maxilla with maxillary alveolus and teeth		Died
2009	4 [14]	Ballistic trauma	France	27, m	N/A	All perioral muscles, maxilla, mandibula, parotid gland, facial nerves		Alive
2009	5 [15]	Burn injury	US	59, m	60, m	Midface soft tissue, maxilla		Died
2009	6 [14]	Ballistic trauma	France	33, m	N/A	Lower two-thirds of facial soft tissue, nasal bone, orbital floor, hard palate, maxilla, mandibula		Alive
2009	7 [16]	Road traffic accident followed by necrotizing inflammation	Spain	42, m	m	Facial soft tissue, tongue, floor of the mouth, salivary glands, mandibula		Died
2009	8 [17]	Explosion injury	France	27, m	N/A	Lips, cheeks, chin, mandibula		Alive
2011	9 [18]	Ballistic trauma	Spain	30, m	41, m	Nose, lips, orbita, lacrimal apparatus, medial canthus ligaments, maxilla, zygomaticum, mandibula		Died
2011	10 [19]	Burn injury	US	25, m	48, m	Forehead soft tissue, orbita, nose, cheeks, and lips		Alive
2011	11 [20]	Balistic trauma	France	45, m	N/A	Nasal bone, mandibula, maxilla		Alive
2011	12 [20]	Balistic trauma	France	41, m	N/A	Nasal bone, mandibula, maxilla		Died
2011	13 [19]	Burn injury	US	30, m	31, m	Forehead soft tissue, eyelids, nose, cheeks, and lips		Alive
2011	14 [19]	Animal attack	US	57, f	42, f	Forehead, eyelids, nose, lips, maxilla, mandibula		Alive
2011	15 [21]	Balistic trauma	Belgium	54, m	22, m	Midface soft tissue, eyelids, orbita, maxilla, mandibula, facial nerve		Alive
2012	16 [22]	Balistic trauma	Turkey	19, f	27, f	Midface soft tissue, nose, upper lip, maxilla, seven teeth		Died
2013	17 [23]	Balistic trauma	Turkey	26, m	42, m	Nasal bone, mandibula, maxilla		Alive
2014	18 [19]	Balistic trauma	US	39, m	40, m	Nose, cheeks, lips, maxilla, mandibula		Alive
2014	19 [24]	Road traffic accident followed by necrotizing inflammation	US	44, m	21, m	Upper two thirds of facial soft tissue, nasal bone, perinasal sinuses, sinunasal mucosa, mandibula, oral cavity, facial nerve, anterior two-thirds of scalp		Alive
2014	20 [25]	Balistic trauma	US	33, m	N/A	Upper and lower lips, tongue, maxilla, mandibula, nasal and nasoethmoidal structures, orbita		Alive
2016	21 [6]	Balistic trauma	Finland	34, m	N/A	Midface soft tissues, nose, maxilla, mandibula, lip and buccal mucosa, oral cavity, tounge, facial, hypoglossal, buccal, supraorbital, infraorbital and mental nerves		Alive
2017	22 [26]	Balistic trauma	US	21, f	31, f	Mandibula, hard palate, glabella, nose, tongue, bilateral facial nerves, eyelids, lips, mimetic muscles		Alive
2018	23 [27]	Balistic trauma	US	25, m	23, m	Nasal bone, zygomaticum, mandibula, maxilla		Alive
2018	24 [6]	Balistic trauma	Finland	58, m	N/A	Total facial soft tissue, maxilla, mandibula		Alive
2018	25 [28]	Balistic trauma	Canada	64, m	Younger than recipient, m	Lower two thirds of facial soft tissue, maxilla, mandibula, nose		Alive

**Table 2:** Summary of all data on post-FT oral health, including oral examination, dentition, dental treatments, dental rehabilitation, occlusion, and mucosal condition

Patient	Length of follow-up (y: year, m: month)	Oral examinations	Dentition	Dental treatments	Dental Rehabilitation	Occlusion	Mucosal Condition
1	10 y	Biopsies of oral mucosa	Patient kept complete healthy dentition	N/A	N/A	N/A	Banff grade III mucosal rejection 94 months post-FT
2	24 m	Biopsies of skin and oral mucosa	N/A	N/A	Dental titanium implants 10 months post-FT	N/A	day 64: Banff grade II mucosal rejection at 64 days post-FT; Banff grade I mucosal rejection until 10 months post-FT
3	8 m	Biopsies of oral mucosa	Incisors/alveolus transplanted with maxilla, all other maxillary teeth cautiously removed because of poor dentition	N/A	N/A	N/A	Banff grade III/IV mucosal rejection at 47 days post-FT
4	19 m	N/A	Maxillar dentition Dd. 4–13, harvested via modified Wasmund technique	N/A	N/A	N/A	N/A
5	3 y	Clinical testing for feeling of full or empty mouth and cold-warm-distinction	Donor teeth were transplanted	Donor teeth from maxilla removed due advanced decay within 6 months post-FT	Additional dental implants post FT, successfully osseointegrated, implant placement in maxilla and mandibula for future dental prostheses	Edentulous	N/A
6	14 m	N/A	Maxillar dentition Dd. 4–13, harvested via modified Wasmund technique, mandibula with dentition	N/A	Cortically anchored disk deisgn implants at one year post-FT	Malocclusion requiring correction, BSSO at 8 months post-FT	N/A
7	16 m	N/A	Upper dentition was removed, all teeth of the lower dentition transplanted from donor	Extraction of upper dentition	Planning of upper prosthetic denture	Malocclusion requiring correction, limited mandibular excursion (10 mm)	N/A
8	53 m	N/A	Receipients maxilla was intact, edentulous mandibula was transplanted	N/A	N/A	N/A	Vacuolization of the basal epithelial cell layer, EBV coinfection of mucosa
9	6 w	Postoperative cepahlometry	Teeth transplanted from donor allograft		N/A	Incomplete occlusion requiring correction, LeFort I osteotomy	N/A
10	39 m	Ten test for intraoral sensation, biopsies of oral mucosa, saliva flow	Edentulous	N/A	Additional dental implants post FT	N/A	Constricted mucosa with rejection of the oral mucosa
11	5 y	Clinical examination, biopsies of oral mucosa, electromyography, viral serologies	Teeth transplanted from donor allograft	Teeth removal one year post-FT	N/A	Malocclusion requiring correction	N/A
12	3.5 y (suicide)	Clinical examination, biopsies of oral mucosa, electromyography, viral serologies	Teeth transplanted from donor allograft, teeth avulsion	N/A	Dental implants	Malocclusion requiring correction	N/A
13	38 m	N/A	No transplanted teeth	N/A	N/A	Angle Class I occlusion	N/A
14	37 m	Ten test for intraoral sensation, mucosal constriction, mucosal rejection, saliva flow, pulse oximeter, nerve testing	Maxillary teeth transplanted	N/A	Retained transplanted teeth, additional five dental implants (one out of five osseointegrated poorly), implant supported lower denture fabricated	Angle Class I occlusion	Mucosal rejection, prominent white lichen planus-like reticular changes
15	3 y	Clinical examination, biopsies of oral mucosa	Maxillary teeth transplanted	N/A	N/A	Angle Class II malocclusion	Acute graft rejection 4 months post-FT
16	2.5 y	Intraoral inspection, intraoral periapical radiography	Seven teeth (Dd. 6–13), intraoperative extraction of Dd. 6 and 13 for dental arch harmony	caries/composite filling: Dd. 12 (donor) and 14 and 18 (recipient), apical radiolucency 3.5–4 mm: D. 12 (2 canals with inadequate root canal filling), endodontic treatment: Dd. 12, 18	N/A	N/A	N/A
17	47 m	Biopsies of oral mucosa	N/A	N/A	N/A	Malocclusion requiring correction, orthognatic surgery 3 months post-FT	N/A
18	3 m	Clinical and radiographic evaluation of intraoral status, function, teethvitality assessment via EPT, testing of blood flow in teeth via pulse oximetry	Maxillary and mandibular teeth transplanted, EPT testing negative in donor teeth	N/A	Retained transplanted teeth	Angle Class III malocclusion	N/A
19	12 m	N/A	Maxillary teeth transplanted, decaying mandibular teeth	Extraction of mandibular teeth due to caries	Dental mandibular implant 8 months post-FT	Angle Class I occlusion, bilateral degenerative changes, corrected by left total condylectomy 6 months pot-FT	N/A
20	1 y	N/A	N/A	N/A	N/A	Angle Class I occlusion	N/A
21	4 y	Clinical and radiologic routine oral examinations, periodontal diagnosis, active matrix metalloproteinase-8 test, biopsies of gingival overgrowths, microbial samples from deep periodontal pockets, diagnostic and/or therapeutic sialoendoscopy, electroneuromyography	Transplanted teeth: Dd. 2–15, 16–19, K, 21–28, T, 30–31, current dentition (04/2020): Dd. 4–16, 19, K, 21–28	Dd. 5, 8, 23–24: endodontic treatments, fillings with composite resin or glass ionomer cement, Dd. 2–4, 15, 18, 23–24, T, 30–31: extracted (caries lesion/periodontits)	No implants placed, prosthetic rehabilitation is planned	N/A	Oral candidiasis, ulcer caused by mastication
22	5 y	Biopsies of oral mucosa	Transplanted maxillary and mandibular teeth	Removal of right second molar and wisdom tooth	N/A	Angle Class III malocclusion	Banff grade II
23	249 d	N/A	Donor dentition was copied via dental splint (presurgical dental impression), all maxillary and mandibular teeth transplanted	N/A	N/A	Left mandibular nonunion, ORIF with left coronoidectomy	N/A
24	2 y	Clinical and radiologic routine oral examinations, periodontal diagnosis, active matrix metalloproteinase-8 test, biopsies of gingival overgrowths, microbial samples from deep periodontal pockets, diagnostic and/or therapeutic sialoendoscopy, electroneuromyography	Transplanted teeth: Dd. 2–3, 6–11, 14, 20–29, current dentition (04/2020): Dd. 3, 6–11, 14, 20–28	Fillings with composite resin or glass ionomer cement, Dd. 2, 29: extracted (caries lesion/periodontits)	Bone level implants placed : 4, 5, 12, 13, 29, 30, successfully osseointegrated	Cross-bite in the left lateral incisor and canine regions, corrected with osteotomy at 15 months post-FT	Oral candidiasis, ulcer caused by mastication
25	3 y	Clinical examination of functional mouth opening	N/A	N/A	Usage of palatal splint and dental occlusion retainer during first year post-FT	Severe anterior open bite, BSSO due to hardware loosening and nonunion	N/A

### Oral examinations

All of the assessed oral examination techniques were conducted postoperatively. The most common oral examination procedure was a biopsy of the oral mucosa (*n* = 11, 44%) to assess for histological signs of tissue rejection. Clinical examination of the oral cavity was conducted in nine (36%) cases, while radiologic examination was performed in five (20%) cases. The Ten Test for assessing intraoral sensation as well as saliva flow in milliliters per minute was each performed in two (8%) patients [19]. For evaluating tooth vitality, pulse oximeter (*n* = 2, 8%), nerve testing (*n* = 1, 4%), and electric pulp testing (EPT) (*n* = 1, 4%) were utilized. Periodontal diagnosis, active-matrix metalloproteinase-8 (aMMP-8) testing, and microbial sampling from deep periodontal pockets were investigated in two (8%) cases, all within a single study.

### Dental occlusion

Across all reviewed studies, a total of 12 (48%) cases with malocclusion were identified. Four (16%) cases reported post-FT occlusion as Angle Class I. In one (4%) study, Angle Class II occlusion was noted, while two (8%) reported Angle Class III occlusion. One (4%) case report each documented a severe anterior open bite, a left mandibular nonunion, and a left-sided cross-bite. Revision surgery to correct maxillomandibular occlusion was reported in nine (36%) cases. Limited maximum mouth opening distance following FT was observed in one (4%) case. Another case report (*n* = 1, 4%) described a total condylectomy to address bilateral degenerative changes in the TMJ six months after the initial FT.

### Dental complications

In 12 (48%) cases, maxillary dentition was transplanted from the donor, while mandibular dentition was transplanted in seven (28%) cases. Among the case reports involving transplanted upper and lower jaw dentition, seven (28%) reported post-surgical tooth extraction. The time frame for tooth removal post-FT ranged from six months to five years. The most common reasons for tooth extraction were caries (*n* = 4, 16%) and periodontitis (*n* = 2, 8%). Post-FT dental treatments were mentioned in eight (32%) cases, with composite and glasionomer fillings performed in three (12%) cases and root canal treatment in two (4%) cases, all involving transplanted donor teeth.

### Mucosal complications

Mucosal rejection signs were reported in seven (28%) FT case reports. The most common (*n* = 4, 16%) parameter for assessment of skin and mucosal rejection was the Banff classification, with Banff grade III rejection reported in two (8%) cases and Banff grade IV in one (4%) case at various times post-FT. Originally developed for acute rejection in kidney transplants, the Banff classification has since been adapted for acute T-cell-mediated rejection in skin-containing tissue transplants [29]. Mucosal constriction affecting TMJ function was reported in one (4%) FT case. Additionally, Lichen planus-like reticular changes in the oral mucosa were observed in one (4%) case. Mucosal involvement with candidiasis and oral ulcers was reported in one study, encompassing two (8%) FT cases. Detailed information on functional outcomes, oral sensation, and post-FT diet are presented in Table 3.

**Table 3:** Summary of post-FT functional outcomes of the oral cavity, oral sensation, and patient diet. All functional outcomes were assessed at the end point of the respective follow-up, unless otherwise stated

Patient	Functional outcome of the oral cavity	Oral sensation	Patient diet
1	Upper lip disfigurement, severely impaired mouth opening, slurred speech	N/A	Eating and drinking were compromised as lip closure was impossible
2	Able to speak by day 10	Clear sensory reinnervation of the grafted skin, for both thermal and mechanical sensations at 3 months post-FT	Able to eat by day 10
3	Smelling, tasting, speaking	Regained after 5 months, discrimination of 7 mm two-point is possible	Drinking from a cup and eating solid foods
4	N/A	Only when deep pressure with mechanical stimulation is applied	N/A
5	Normal oral competence, improved speech	In 92% of allograft skin and oral mucosa sensation at 0.07 g of pressure, intraoral sensation was 8/10	Able to eat normally
6	Patient is well understood in a face-to-face interview	N/A	Able to eat a normal diet by mouth
7	Mandibular excursion is 10 mm	N/A	Able to swallow
8	Phonation is preserved, movements of perioral area are limited	Transient decrease in sensitivity, improving from one year post-FT onwards	Eating and drinking is not compromised
9	Regained muscle-movement	Regained total sensation in cheeks and intraoral mucosa, no sensation in the lips	Solid diet
10	Improved oral competence, speech, smell and nasal breathing, reduced mouth opening of 23 mm	N/A	N/A
11	Limited speech	N/A	N/A
12	Limited speech	N/A	N/A
13	Improved oral competence and speech	N/A	N/A
14	Restoration of oral competence, improved speech	Return of sensation three months post-FT, intraoral sensation was 7/10	N/A
15	Mouth opening of 4 cm, from one month post-FT onwards discrete muscle activity in upper and lower lip	Sensation evolved from 3 months post-FT onwards, patient felt tingling until 31 months post-FT	Oral intake is considered normal at 2 months post-FT
16	N/A	Via electric pulp test sensory response of teeth was ascertained	N/A
17	N/A	Satisfactory sensation at four moths post-FT	N/A
18	Oral competence, improved speech	Intraoral sensation was 1/10	Patient was successfully decannulated and the feeding tube removed
19	Limited motor function with minimal motor recovery, able to smile	Recovered touch sensation of bilateral cheeks, able to localize but no two-point discrimination yet	N/A
20	Improved jaw mobility (11.7 mm), improved mouth opening (1 cm to 3 cm), improved speech intelligibility at one year post-FT	N/A	Patient is eating an unrestricted diet
21	Improved mouth opening and lip competence at 48 months post-FT	Restored pain and light touch sensation, except discriminatory sensation of less than 16 mm	N/A
22	N/A	N/A	N/A
23	Satisfactory speech intelligibility, muscle weakness when smiling	Sensation to light touch was intact bilaterally	Patients entire nutrition is orally
24	Improved mouth opening, mimetic muscle function and nerve activity of all transplanted nerves	Sensation is improving	Eating and swallowing is impaired
25	N/A	N/A	N/A

### Prosthetic treatment

Dental implants were placed in eight (32%) cases across the reviewed studies. Only two cases provided the date of implantation (eight and ten months post-FT). Osseointegration of these implants was reported in three (12%) cases, although in one case report one out of five implants failed to achieve osseointegration and remained non-functional. Interestingly, one (4%) case report described the use of cortically anchored disk-design implants. One (4%) further study reported data on the design of dental implants, describing the placement of six bone level implants. Among the reviewed articles, four (16%) cases were reported to undergo oral rehabilitation involving prosthetics, of which two (8%) were implant-based.

A narrative review of all included cases is available in the Supplementary Digital Content 5.

**Fig. 2:**
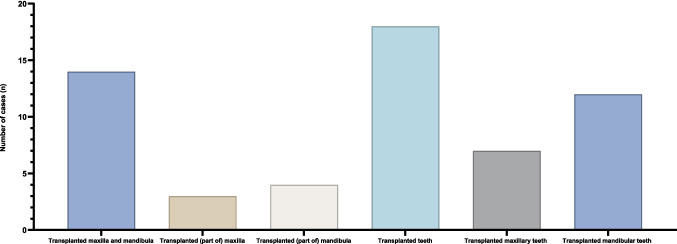
Distribution of cases according to the transplanted dentoalveolar area

**Fig. 3:**
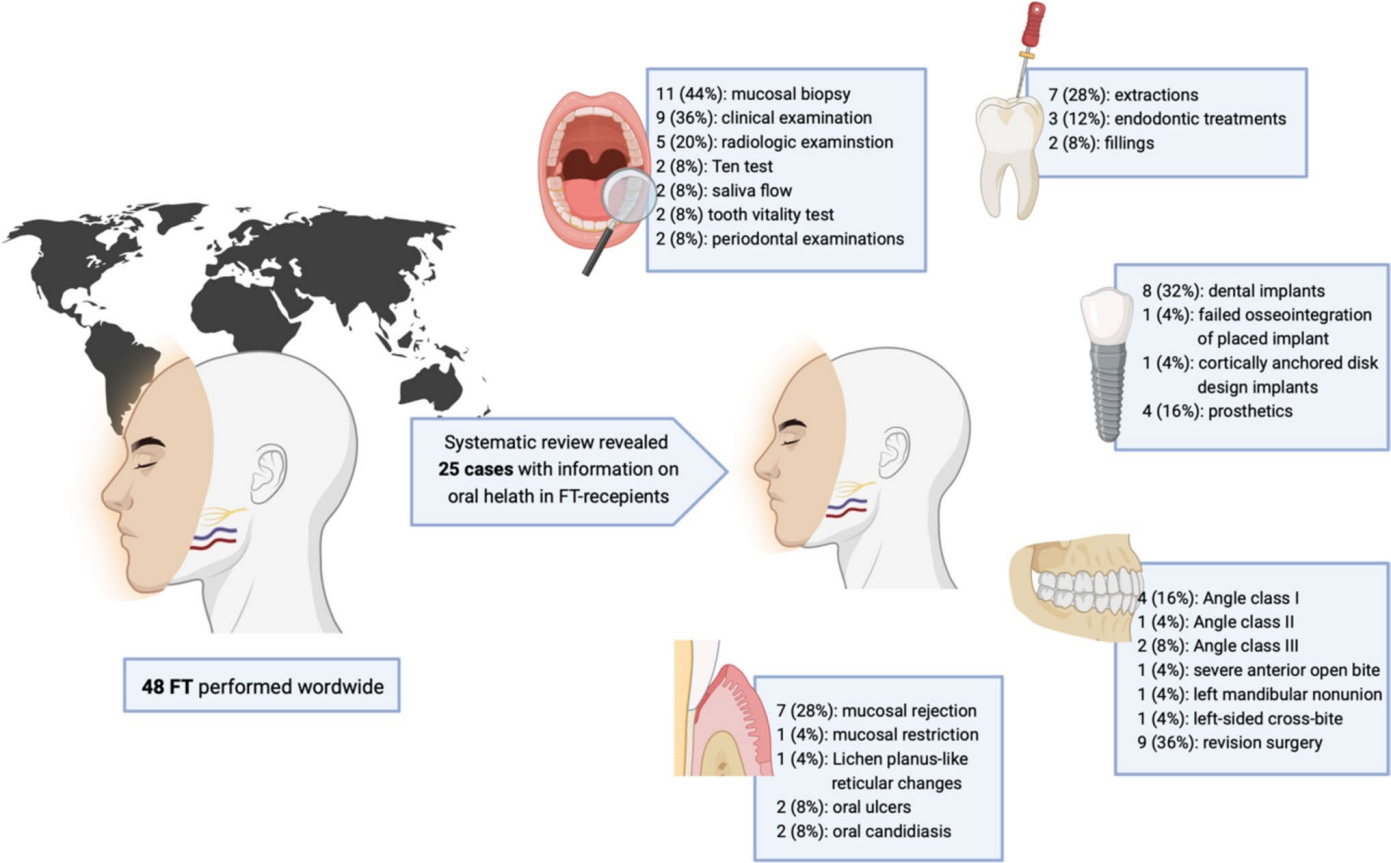
Graphical abstract

Figure 2 displays the distribution of cases according to the transplanted dentoalveolar regions, while Fig. 3 presents a graphical abstract of the study.

## Discussion

FT represents the next stage of facial reconstruction providing an innovative treatment plan for severe (mid-)facial defects. There is a paucity of up-to-date literature that investigates the role of oral health in FT patients. This systematic review aims to fill in this research gap.

### Role of routine oral examinations in FT patients

Across all the reviewed studies, only 17 reported conducting oral examinations post-FT, and none documented a preoperative intraoral screening of the donor’s oral health. Notably, there was no standardized protocol for oral examinations pre- and post-FT, leading to an inconsistent and heterogeneous set of examination techniques across the studies reviewed. A common procedure in FT patients involved regular biopsies of the oral mucosa. These biopsies served less as a routine oral assessment and more as an easily accessible site for testing histological signs of tissue rejection. Additionally, dental screenings included tests for dental vitality, such as pulse oximeter and electric pulp testing, which are particularly valuable in evaluating transplanted teeth. However, caution is advised when interpreting the results of dental nerve testing in transplanted teeth, as nerve regeneration in the pulp can take at least two months, and potentially longer in FT recipients. Only one study reported on comprehensive dental examinations, which included assessments of periodontal condition, microbial sampling from deep periodontal pockets, and active matrix metalloproteinase-8 (aMMP-8) testing, a biomarker for diagnosing periodontal and peri-implant diseases [6]. However, there is a body of evidence indicating that (solid) organ transplant recipients require routine examination of oral health, starting before the transplantation surgery. Early diagnosis of caries or periodontitis through radiologic imaging and clinical examinations may prevent potential sources of oral infection that could lead to systemic infections post-transplantation [30]. Notably, studies suggest that localized infections originating from oral sources can negatively impact the overall prognosis of the transplant [31]. 

Given that eight FT recipients required tooth extractions and other dental treatments post-FT, we emphasize the importance of routine oral examinations in these patients. In line with the recommendations of Özel et al., we advocate for a thorough preoperative screening of the donor’s intraoral condition and dental status [22]. This should be considered before transplantation, as it may necessitate preoperative tooth extractions and prosthetic planning to ensure adequate occlusion. Moreover, regular and standardized postoperative oral examinations may help maintain oral health and promptly address any intraoral infections, particularly in this highly immunosuppressed patient population.

### Dental treatments in FT patients

In nine of the reviewed FT patients, different dental treatments (e.g., extraction, filling, or root canal treatment) were performed. A commonly reported reason for the poor dental condition of these patients was the suboptimal oral status of the donor. Furthermore, FT patients have a higher need for an infection-free oral environment due to the lifelong high-dose immunosuppression they receive [32]. In their article on dental treatments following organ transplantation, Fabuel et al. recommended intensive education on oral hygiene, dietary improvements, the removal of prosthetics and orthodontics, and infection prevention during the first three months post-FT. After three months post-transplantation, elective dental treatments can be safely performed, with six months post-surgery being considered the optimal time for such procedures. For more invasive treatments, the administration of prophylactic antibiotics and a preoperative blood count are recommended [33]. Özel et al. provided a detailed protocol of a root canal treatment performed on an FT patient one month after surgery. The procedure was carried out using standard protocols, including the use of 2.5% sodium hypochlorite as a rinsing solution and gutta-percha with AH-26 sealer for the filling. Additionally, two other non-transplanted teeth in the same patient were also treated with root canal fillings. Remarkably, the healing process was uncomplicated, likely due to the effective reconstructed blood circulation in the transplanted part of the maxilla [22]. Similarly, in other case reports, root canal treatments of transplanted teeth were performed successfully [6]. In FT patients, preventing systemic and intraoral infections is crucial. Physicians often have to choose between conservative dental treatments, which carry a higher risk of persistent local infection, and tooth extraction. Given the complexity of these cases, dental treatment decisions should be made on a case-by-case basis, reviewing the patient’s overall health status, current medications, and postoperative comorbidities. Therefore, we emphasize the importance of interdisciplinary collaboration among transplant physicians, facial surgeons, and dentists.

### Strategies for dental rehabilitation

Eleven FT patients presented with a (partly) edentulous maxilla, mandibula, or both, often due to the donor’s preoperative edentulous condition or the removal of teeth postoperatively. In these cases, oral rehabilitation becomes a critical step in improving the patient’s quality of life by restoring oral functions such as speaking, mastication, and aesthetic appearance. To achieve oral rehabilitation and secure intraskeletal fixation, dental implants have been placed in seven FT patients. These implants have been inserted not only into the recipient’s native jaw but also into transplanted parts of the maxilla and mandible [34]. Studies on dental implants in transplanted patients report a high rate of osseointegration, attributed to consistent immunosuppressive therapy [35]. Of the reviewed studies, only one implant failed to osseointegrate correctly and remained functionless. Notably, this patient had received four dental implants in the recipient’s native mandible at the time of the FT surgery [19]. Given the limited number of FT performed to date, clinical evidence regarding the success rate of dental implants in FT patients is currently lacking. However, there is a substantial body of research investigating implant success rates in autogenous free flaps (AFF), such as free fibula flaps (FFF) and free scapular flaps [36]. These studies report a success rate of approximately 94–100% for dental implants placed in FFF [37–39]. Some studies also suggest that secondary implantation, rather than immediate implant placement during the FT procedure, may be preferable. This preference is due to the additional operation time and the lack of thorough planning for long-term dental rehabilitation, such as prosthetic alignment, during the initial FT procedure [38]. In four of the reviewed studies, oral rehabilitation using prostheses was described. However, detailed information on the fabrication of prosthetic dentures specifically for FT patients is lacking. Adapting prostheses to the alveolar ridge in AFF-reconstructed areas can be more complex compared to FT cases. In AFF patients, debulking the soft tissue component and adequately shaping the flap are essential for securing removable prostheses. Conversely, in FT reconstructions, the alveolar ridge is typically already well-contoured, which may potentially simplify the prosthetic fitting process [40]. 

### Challenging malocclusion following FT

FT can be performed in various configurations: (i) without skeletal involvement of the maxilla or mandible, (ii) involving only the maxilla (in part or whole), (iii) involving only the mandible (in part or whole), or (iv) involving both the maxilla and mandible. Postoperative malocclusion, such as Angle Class II or III, is a commonly reported complication, particularly when both the maxilla and mandible are transplanted [21, 26]. However, even in cases where only one jaw is transplanted, malocclusion can still occur [16]. Achieving a well-functioning occlusal plane, where the transplanted segment aligns well with the recipient’s anatomy in three dimensions, is challenging in the three later scenarios. When both native and transplanted jaws contribute to occlusion, discrepancies can arise due to mismatches between the recipient’s and donor’s skeletal dimensions [19] Conversely, when both jaws are transplanted simultaneously, the challenge is to create a new alignment without any natural reference points. Minimal misalignments can often be corrected with postoperative orthodontic treatment [6]. However, significant malocclusions typically require orthognathic surgery, such as LeFort I osteotomy or bilateral sagittal split osteotomy [14]. To address these challenges, modern technologies such as presurgical virtual planning and intraoperative guidance have been introduced. Using 3D-printed cutting guides and intraoperative dynamic navigation allows for more accurate positioning of the maxillary and mandibular bone segments [41]. Furthermore, employing a CAD/CAM-fabricated splint followed by intermaxillary fixation can help establish the correct maxilla-mandible relationship intraoperatively. This approach is especially beneficial in dentate patients but may also reduce the need for secondary surgeries.

### Conditions of the oral mucosa

Although FT patients receive ongoing immunosuppressive therapy, the majority experience acute graft rejection, particularly within the first year post-transplantation. Rejection of the oral mucosa is a common issue, exacerbated by the side effects of immunosuppressive medications such as tacrolimus and mycophenolate mofetil, which can manifest as ulcers or erythema [42, 43]. Additionally, oral lichenoid interface mucositis and asymptomatic reticular lichenoid lesions may develop, typically treated with topical glucocorticoids [32]. Despite several reviewed studies recommending routine oral biopsies to histologically detect graft rejection, there is ongoing debate regarding their utility in FT patients [44, 45]. Some studies suggest that the oral mucosa is more antigenic, and that low-dose, daily trauma from routine activities could lead to histological misinterpretations [46, 47]. Long-term, high-dose immunosuppression also increases the risk of fungal infections. In FT patients with oral candidiasis, strains of *Candida albicans* and *Candida glabrata* have been identified. Oral candidiasis can range from asymptomatic white lesions to clinical manifestations such as mucosal inflammation, burning, erythema, dysgeusia, and hyperplasia of the oral mucosa. Treatment typically involves antifungal medications like fluconazole and nystatin. [34, 48] Thermal and mechanical sensation in the intraoral mucosa was assessed in five of the included cases. In four out of five FT cases, intraoral sensation was reasonably restored, although sensory nerve rehabilitation can take several months. Sensory testing, including the Ten Test, sharp versus dull testing, and thermal sensation assessments, provided partial comparability across different cases. However, we observed a strong heterogeneity in sensory testing methods, with a lack of standardized scoring systems (e.g., the Ten Test), making it challenging to compare neuronal recovery across different FT cases [49]. Since oral sensation is crucial for functions such as lip competence while drinking and eating, swallowing, and differentiating between hot and cold food, this study underscores the importance of not only reconstructing motor nerves during FT surgery but also ensuring the restoration of mucosal sensory fibers. This study, therefore, highlights the need for standardized scoring systems for mucosal sensory assessment to be integrated into routine oral examinations. As such, additional scoring systems that include oral health parameters for pre- and post-surgical evaluation of FT patients and donors may represent an area of future research.

### Limitations

The conclusions of this systematic review should be interpreted with caution due to several limitations. The exclusive reliance on case reports and case series may introduce bias and affect the reliability of conclusions regarding oral health conditions in FT patients. Additionally, the limited number of included articles (19 out of 6,984 studies screened), along with the small number of FT procedures performed to date, may restrict the applicability and generalizability of the findings. Moreover, most studies lack detailed information on oral status, such as International Caries Detection and Assessment System classification, periodontitis grading, or exact post-FT dentition, as well as specifics on implant design, material, and brand. Furthermore, the lack of standardized guidelines for evaluating oral health in FT patients limits the transferability of the results.

## Conclusion

This systematic review underscores the need for comprehensive preoperative and routine postoperative oral assessments in FT patients, given the challenges of immunosuppression and tissue rejection. While successful dental treatments and rehabilitation have been reported, standardized guidelines for oral care in FT patients are still warranted. The complexity of achieving adequate occlusion and managing oral mucosal health highlights the importance of cross-disciplinary efforts. Advancements in surgical technology, such as virtual planning and dynamic navigation, may enhance outcomes in dental rehabilitation and occlusal alignment.

## Supplementary Information

Below is the link to the electronic supplementary material.

ESM 1 (DOCX 99.2 KB)

## Data Availability

No datasets were generated or analysed during the current study.
