# Supplementary material for: Oral health and rehabilitation in face transplant recipients – a systematic review
Source: Clin Oral Investig. 2025 Jan 6;29(1):47. doi: 10.1007/s00784-024-06078-3 (PMC11703954; doi:10.1007/s00784-024-06078-3)
Supplement: Supplementary file 1 — (DOCX 99.2 KB) [file 784_2024_6078_MOESM1_ESM.docx]

Supplemental Appendix:

Supplement to: Knoedler, , et. al. “From Novel Facial Measurements to Facial Implantology – A systematic review”

Contents:

Supplement digital content 1
Supplement digital content 2
Supplement digital content 3
Supplement digital content 4
Supplement digital content 5

**Supplement digital content 1**. Search strings in PubMed/MEDLINE, Web of Science, EMBASE, and CENTRAL databases with the respective number of search results.

| Database | Search String | Results |
| --- | --- | --- |
| PubMed/MEDLINE | ("facial"[Title/Abstract] OR "face"[Title/Abstract])  AND  ("transplant"[Title/Abstract] OR "VCA"[Title/Abstract] OR "vascularized composite allograft"[Title/Abstract] OR "vascularized composite allotransplantation"[Title/Abstract] OR "allograft"[Title/Abstract] OR "implant"[Title/Abstract])  AND  ("oral health"[Title/Abstract] OR "oral rehabilitation"[Title/Abstract] OR "oral care"[Title/Abstract] OR "intraoral"[Title/Abstract] OR "dental implants"[Title/Abstract] OR "prosthesis"[Title/Abstract]) | 2,392 |
| Web of Science | TS=("facial" OR "face")  AND  TS=("transplant" OR "VCA" OR "vascularized composite allograft" OR "vascularized composite allotransplantation" OR "allograft" OR "implant")  AND  TS=("oral health" OR "oral rehabilitation" OR "oral care" OR "intraoral" OR "dental implants" OR "prosthesis") | 1,058 |
| EMBASE | ('facial':ti,ab OR 'face':ti,ab)  AND  ('transplant':ti,ab OR 'VCA':ti,ab OR 'vascularized composite allograft':ti,ab OR 'vascularized composite allotransplantation':ti,ab OR 'allograft':ti,ab OR 'implant':ti,ab)  AND  ('oral health':ti,ab OR 'oral rehabilitation':ti,ab OR 'oral care':ti,ab OR 'intraoral':ti,ab OR 'dental implants':ti,ab OR 'prosthesis':ti,ab) | 1,591 |

| CENTRAL | ("facial" OR "face")  AND  ("transplant" OR "VCA" OR "vascularized composite allograft" OR "vascularized composite allotransplantation" OR "allograft" OR "implant")  AND  ("oral health" OR "oral rehabilitation" OR "oral care" OR "intraoral" OR "dental implants" OR "prosthesis") | 1,943 |
| --- | --- | --- |

**Supplement digital content 2.** General data on all performed FT worldwide.

| **Patient** | **Year of face transplant** | **Country** | **Reason for facial defect** | **Recipient age** | **Recipient gender (f: female, m: male)** | **Donor age** | **Donor gender (f: female, m: male)** | **Length of follow-up (y: year, m: month, w: week, d: day)** | | **Reconstructed areas** | **Immunosuppression (TGB: thymoglobulin, TAC: tacrolimus, MMF: mycophenolate mofetil, PDN: prednisolone)** | | **Information on oral status availabe** |
| --- | --- | --- | --- | --- | --- | --- | --- | --- | --- | --- | --- | --- | --- |
| 1^1^ | 2005 | France | Animal attack | 38 | f | 46 | f | 10 y | Nose, lips, chin, cheeks | | | TGB, TAC, MMF, PDN | Yes |
| 2^2^ | 2006 | China | Animal attack | 30 | m | 25 | m | 24 m | Nose, upper lip, maxillary sinus, orbita, zygomaticum, parotid gland | | | TAC, MMF, CST, humanized IL-2R antibody | No |
| 3^3^ | 2007 | France | Neurofibromatosis | 29 | m | N/A | N/A | 24m | Facial soft tissue | | | TGB, TAC, MMF, PDN | Yes |
| 4^4^ | 2008 | US | Ballistic trauma | 45 | f | Age matching | f | 8 m | Midface soft tissue, nose, cheeks, upper lip, right globe, maxilla with maxillary alveolus and teeth | | | TGB, TAC, MMF, CST | Yes |
| 5^5^ | 2009 | France | Ballistic trauma | 27 | m | N/A | N/A | 19 and 14 m | All perioral muscles, maxilla, mandibula, parotid gland, facial nerves | | | TGB, TAC, MMF, PDN, extracorporal photopheresis | Yes |
| 6^6^ | 2009 | France | Burn injury | 37 | m | 59 | m | 35 d | Facial soft tissue, lower lip, scalp | | | TGB, TAC, MMF, PDN | No |
| 7^7^ | 2009 | US | Burn injury | 59 | m | 60 | m | 3 y | Midface soft tissue, maxilla | | | TGB, TAC, MMF, CST | Yes |
| 8^5^ | 2009 | France | Ballistic trauma | 33 | m | N/A | N/A | 14 m | Lower two-thirds of facial soft tissue, nasal bone, orbital floor, hard palate, maxilla, mandibula | | | TGB, TAC, MMF, PDN | Yes |
| 9^8^ | 2009 | Spain | Road traffic accident followed by necrotizing inflammation | 42 | m | N/A | m | 16 m | Facial soft tissue, tongue, floor of the mouth, salivary glands, mandibula | | | TAC, MMF, CST, Basiliximab, antiretroviral modified calcineurin inhibitors | Yes |
| 10^9^ | 2009 | France | Explosion injury | 27 | m | N/A | N/A | 3+ y (1 year post operatively and yearly afterwards) | Lips, cheeks, chin, mandibula | | | TGB, TAC, MMF, CST | Yes |
| 11^10^ | 2010 | Spain | Neurofibromatosis | 35 | m | N/A | N/A | 6 m | Lower third of facial soft tissue, lips, cheeks, chin, all perioral muscles, facial nerve, V2, V3, parotid gland | | | TAC, MMF, steroids (not further specified) | No |
| 12^11^ | 2011 | Spain | Ballistic trauma | 30 | m | 41 | m | 6 w | Nose, lips, orbita, lacrimal apparatus, medial canthus ligaments, maxilla, zygomaticum, mandibula | | | TGB, TAC, MMF, PDN | Yes |
| 13^12^ | 2010 | France | Neurofibromatosis | 35 | m | N/A | N/A | 9.2 y | Nasal bone, mandibula, maxilla | | | TGB, TAC, MMF, PDN, extracorporal photopheresis | No |
| 14^13^ | 2011 | US | Burn injury | 25 | m | 48 | m | 39 m | Forehead soft tissue, orbita, nose, cheeks, and lips | | | TAC, MMF, steroids (not further specified) | Yes |
| 15^12^ | 2011 | France | Ballistic trauma | 45 | m | N/A | N/A | 5 y | Nasal bone, mandibula, maxilla | | | TGB, TAC, MMF, PDN, extracorporal photopheresis | Yes |
| 16^12^ | 2011 | France | Ballistic trauma | 41 | m | N/A | N/A | 3.5 y (suicide) | Nasal bone, mandibula, maxilla | | | TGB, TAC, MMF, PDN, extracorporal photopheresis | Yes |
| 17^13^ | 2011 | US | Burn injury | 30 | m | 31 | m | 38 m | Forehead soft tissue, eyelids, nose, cheeks, and lips | | | TGB, TAC, MMF, PDN | Yes |
| 18^13^ | 2011 | US | Animal attack | 57 | f | 42 | f | 37 m | Forehead, eyelids, nose, lips, maxilla, mandibula | | | TGB, TAC, MMF, PDN | Yes |
| 19^14^ | 2011 | Belgium | Ballistic trauma | 54 | m | 22 | m | 3 y | Midface soft tissue, eyelids, orbita, maxilla, mandibula, facial nerve | | | TGB, TAC, MMF, PDN | Yes |
| 20^15^ | 2012 | Turkey | Burn injury | 19 | m | 37 | m | 65 m | Total facial soft tissue | | | TGB, TAC, MMF, PDN | No |
| 21^16^ | 2012 | Turkey | Burn injury | 25 | m | N/A | N/A | 72 m | Total facial soft tissue, nose, lips, labial mucosa, left ear | | | TGB, TAC, MMF, PDN | No |
| 22^17^ | 2012 | Turkey | Ballistic trauma | 20 | f | 27 | f | 2.5 y | Midface soft tissue, nose, upper lip, maxilla, seven teeth | | | Immunomodulator FK506, further immunosuppression not specified | Yes |
| 23^18^ | 2012 | US | Ballistic trauma | 37 | m | 21 | m | N/A | Total facial soft tissue, maxilla, mandibula, nasal bone, zygomaticum, tongue | | | PDN, TAC, MMF, steroids (not further specified), Alemtuzumab | No |
| 24^15^ | 2012 | Turkey | Burn injury | 35 | m | 19 | m | mean of 53 m | Total facial soft tissue and musculature, eyelids, nasal bone, anterior scalp | | | TAG, TAC, MMF, PDN | No |
| 25^19^ | 2012 | France | - | 52 | f | N/A | N/A | 5 y | Lower and middle floor of the face, tongue | | | N/A | No |
| 26^20^ | 2013 | US | Burn injury | 45 | f | 56 | f | 16 m | Forehead soft tissue, eyelids, nose, cheeks, lips, ears | | | TGB, TAC, MMF, PDN | No |
| 27^21^ | 2013 | Poland | Blunt trauma | 31 | m | 30 | m | 2 y | Nose, maxilla, mandibula, zygomaticum, lips, oral cavity, oro- and nasopharynx | | | TGB, TAC, MMF, PDN | No |
| 28^15^ | 2013 | Turkey | Ballistic trauma | 26 | m | 42 | m | 47 m | Nasal bone, mandibula, maxilla | | | TGB, TAC, MMF, PDN | Yes |
| 29^15^ | 2013 | Turkey | Ballistic trauma | 54 | m | 35 | m | 11 m | Midface soft tissue, nasal bone, mandibula, maxilla, eyelids, tongue, anterior scalp | | | TGB, TAC, MMF, PDN | No |
| 30^22^ | 2013 | Poland | Neurofibromatosis type 1 | 28 | f | 19 | f | 1 y | Full facial soft tissue | | | TGB, TAC, MMF, PDN | No |
| 31^15^ | 2013 | Turkey | Ballistic trauma | 22 | m | 34 | m | 24 m | Nose, upper lip | | | TGB, TAC, MMF, PDN | No |
| 32^13^ | 2014 | US | Ballistic trauma | 39 | m | 40 | m | median 37.5 m, range 3-52 m | Nose, cheeks, lips, maxilla, mandibula | | | TGB, TAC, MMF, PDN | Yes |
| 33^23^ | 2014 | US | Road traffic accident followed by necrotizing inflammation | 44 | m | 21 | m | 12 m | Upper two thirds of facial soft tissue, nasal bone, perinasal sinuses, sinunasal mucosa, mandibula, oral cavity, facial nerve, anterior two-thirds of scalp | | | TGB, TAC, CST, MMF, Solumedrol | Yes |
| 34^24^ | 2014 | US | Ballistic trauma | 33 | m | N/A | N/A | 1 y | Upper and lower lips, tongue, maxilla, mandibula, nasal and nasoethmoidal structures, orbita | | | N/A | Yes |
| 35^25^ | 2015 | Spain | Arteriovenous malformation | 45 | m | N/A | N/A | 5 y | Lower facial soft tissue, neck, lips, tongue, pharynx | | | N/A | No |
| 36^26^ | 2015 | Russia | Burn injury | 22 | m | 51 | m | 4 y | Full facial soft tissue | | | MMF, PDN, Cyclosporin, IL2R-antagonist | No |
| 37^27^ | 2016 | Spain | Burn injury | 41 | m | N/A | N/A | 15 m (?) | Total facial soft tissue, eyelids, ears, scalp and skeletal subunit | | | not further specified | No |
| 38^28^ | 2016 | Finland | Ballistic trauma | 34 | m | N/A | N/A | 4 y | Midface soft tissues, nose, maxilla, mandibula, lip and buccal mucosa, oral cavity, tounge, facial, hypoglossal, buccal, supraorbital, infraorbital and mental nerves | | | TAC, MMF, Medrol | Yes |
| 39^29^ | 2016 | US | Ballistic trauma | 31 | m | N/A | N/A | 20 m | Maxilla, mandibula | | | TGB, TAC, MMF, PDN | No |
| 40^30^ | 2017 | US | Ballistic trauma | 21 | f | 31 | f | 4 y | Mandibula, hard palate, glabella, nose, tongue, bilateral facial nerves, eyelids, lips, mimetic muscles | | | TAC, MMF, PDN | Yes |
| 41^31^ | 2018 | US | Ballistic trauma | 25 | m | 23 | m | up to 1 y | Nasal bone, zygomaticum, mandibula, maxilla | | | TGB, Rituximab | Yes |
| 42^32^ | 2018 | France | Face allograft chronic rejection | 44 | m | N/A | N/A | 30 m | Ttotal facial soft tissue | | | TGB, PDN, Rituximab, Belimumab, Eculizumab, extracorporal plasmapheresis | No |
| 43^28^ | 2018 | Finland | Ballistic trauma | 58 | m | N/A | N/A | 2 y | Total facial soft tissue, maxilla, mandibula | | | TAC, MMF, Medrol | Yes |
| 44^33^ | 2018 | Canada | Ballistic trauma | 64 | m | Younger than recipient | m | 3 y | Lower two thirds of facial soft tissue, maxilla, mandibula, nose | | | TGB, TAC, MMF, PDN | Yes |
| 45^34^ | 2018 | Itlay | Neurofibromatosis | 49 | f | 21 | f | 2 d | Zygomaticum, maxilla, mandibula, oral cavity, facial nerve, infraorbital nerve, nose | | | TGB, TAC, MMF, PDN | No |
| 46^35^ | 2019 | US | Burn injury | 68 | m | N/A | N/A | 13 m | Full facial soft tissue | | | TGB, TAC, MMF, PDN | No |
| 47^36^ | 2020 | US | Face allograft chronic reaction | 52 | f | 36 | f | 8 m | Removal of rejected FT and retransplant of a new full face (same areas as in 2013) | | | TAC, MMF, PDN | No |
| 48^37^ | 2020 | US | Burn injury | 21 | m | 47 | m | 125 d | Full facial soft tissue | | | TGB, TAC, MMF, PDN, Rituximab | No |

**Supplement digital content 3.** NOS quality assessment of the studies included. N/A indicates that the respective data point was not available.

| Study (Chronological Order) | ***Newcastle-Ottawa Scale*** | | | ***Total Sum*** |
| --- | --- | --- | --- | --- |
|  | *Selection* | *Comparability* | *Assessment* |  |
|  | *(maximum 4*)* | *(maximum 2*)* | *(maximum 3*)* |  |
| Morelon et al. (2005) | ** | N/A | ** | 4 |
| Lantieri et al. (2007) | ** | N/A | ** | 4 |
| Siemionow et al. (2008) | ** | N/A | ** | 4 |
| Meningaud et al. (2009) | ** | * | ** | 5 |
| Diaz-Siso et al. (2009) | ** | N/A | ** | 4 |
| Cavadas et al. (2009) | ** | N/A | ** | 4 |
| Petruzzo et al. (2009) | ** | N/A | ** | 4 |
| Barret et al. (2011) | ** | * | ** | 5 |
| Wall et al. (2011) | ** | N/A | ** | 4 |
| Lantieri et al. (2011) | ** | * | ** | 5 |
| Roche et al. (2011) | ** | N/A | ** | 4 |
| Ozel et al. (2012) | ** | N/A | ** | 4 |
| Ozkan et al. (2013) | ** | * | ** | 5 |
| Hashem et al. (2014) | ** | N/A | ** | 4 |
| Krezdorn et al. (2014) | ** | N/A | ** | 4 |
| Norrmann et al. (2016) | ** | * | ** | 5 |
| Knackstedt et al. (2017) | ** | N/A | ** | 4 |
| Kantar et al. (2018) | ** | N/A | ** | 4 |
| Govshievich et al. (2018) | ** | N/A | ** | 4 |

**Supplement digital content 4**. LOE of the studies included.

| Study (Chronological Order) | ***Level of Evidence*** |
| --- | --- |
|  |  |
|  |  |
| Morelon et al. (2005) | IV |
| Lantieri et al. (2007) | IV |
| Siemionow et al. (2008) | IV |
| Meningaud et al. (2009) | IV |
| Diaz-Siso et al. (2009) | IV |
| Cavadas et al. (2009) | IV |
| Petruzzo et al. (2009) | IV |
| Barret et al. (2011) | IV |
| Wall et al. (2011) | IV |
| Lantieri et al. (2011) | IV |
| Roche et al. (2011) | IV |
| Ozel et al. (2012) | IV |
| Ozkan et al. (2013) | IV |
| Hashem et al. (2014) | IV |
| Krezdorn et al. (2014) | IV |
| Norrmann et al. (2016) | IV |
| Knackstedt et al. (2017) | IV |
| Kantar et al. (2018) | IV |
| Govshievich et al. (2018) | IV |

**Supplement digital content 5.** Narrative review of all 18 included studies.

*Overview of the case reports*

Morelon et al. reported on a soft tissue FT conducted in 2005. Ten years post-FT, the patient experienced a Banff grade III rejection, which also affected the oral mucosa, necessitating the surgical removal of the necrotic areas. ^1^ Lantieri et al. published a case report on soft tissue FT, describing the placement of dental titanium implants ten months post-transplant.^3^ Siemionow et al. presented a case report on a near-total FT performed in 2008. All maxillary teeth, except the incisors, were removed preemptively due to the poor condition of the donor's teeth, while the recipient's mandibular dentition was preserved. ^38^ Meningaud et al. described two partial-FT cases, noting that in one case, bilateral mandibular osteotomy was performed eight months post-FT due to unsatisfactory occlusion.^5^ In a three-year follow-up report, Diaz-Siso et al. detailed the outcomes of a midface transplantation performed in 2009. Within 6 months, all teeth of the maxillary donor dentition had to be extracted due to advanced decay.^39^ Cavadas et al. reported on a lower FT that included the mandible, floor of the mouth, and tongue. During the preparatory surgery, the upper dental arch was removed with plans to fit an upper prosthetic denture modeled after the donor's.^8^ In a case report by Petruzzo et al., a FT performed in 2009 was described, where the patient experienced limited mouth opening to approximately 2 cm, with restricted movements in the perioral area due to fibrosis and soft tissue retraction.^9^ Barret et al. discussed secondary procedures in full-face transplant patients, highlighting a case where a patient developed malocclusions characterized by an open bite and posterior molar contact. This issue was addressed with a LeFort I rotational inferior distraction osteotomy.^11^ Reviewing six FT cases performed in the United States, Wall et al. focused on intraoral considerations. The oral examinations conducted included dental nerve testing, pulse oximetry, assessment of lip competence, evaluation of mucosal constriction and rejection, the Ten Test for intraoral sensation, and measurement of unstimulated saliva flow in milliliters per minute.^13^ Roche et al. reported on a FT performed in 2011 on a Belgian patient. Oral examination revealed a class II malocclusion with an overjet of the maxilla, attributed to a size mismatch between the donor maxilla and the recipient mandible.^14^ In a case report by Özel et al., the condition of donor teeth post-FT was examined, with findings that three transplanted teeth developed caries and one had a periapical lesion within one month post-FT. These teeth were subsequently treated with fillings and root canal therapy.^17^ A report on five FT cases for various types of facial disfigurement included a third case where orthognathic surgery was performed three months post-transplant to achieve adequate occlusion.^15^ In a case report by Hashem et al., FT for reconstruction following granulomatosis with polyangiitis was described. Eight months post-FT, all remaining mandibular teeth were extracted due to severe carious lesions.^23^ Krezdorn et al. presented two cases of trismus in FT patients, including one case of severe mandibular movement limitation due to extensive scarring and bilateral fibrotic TMJ dysfunction.^24^ Norrmann et al. reported on two FT conducted in Finland. Oral examinations for these cases included periodontal diagnosis, active matrix metalloproteinase-8 (aMMP-8) testing, biopsies of gingival overgrowths, and microbial sampling from deep periodontal pockets.^28^ Knackstedt et al. presented the case of an 18-year-old woman who underwent FT. 14 months post-FT, a bilateral sagittal split osteotomy with a mandibular setback of 7 mm was performed to address her Class III malocclusion.^30^ In a study by Kantar et al., an FT was performed where the transplanted dentition was positioned within a prefabricated, three-dimensionally printed dental splint, with fixation achieved using preplaced maxillary and mandibular skeletal anchorage screws.^31^ In a case report by Govshievich et al., a FT patient experienced inadequate oral closure postoperatively, characterized by a 1 cm residual lip incompetence during forceful mouth closure.^33^

Literature

1. Morelon E, Petruzzo P, Kanitakis J, et al. Face Transplantation: Partial Graft Loss of the First Case 10 Years Later. Am J Transplant 2017: 17: 1935-40.

2. Guo S, Han Y, Zhang X, et al. Human facial allotransplantation: a 2-year follow-up study. Lancet 2008: 372: 631-8.

3. Lantieri L, Meningaud J-P, Grimbert P, et al. Repair of the lower and middle parts of the face by composite tissue allotransplantation in a patient with massive plexiform neurofibroma: a 1-year follow-up study. The Lancet 2008: 372: 639-45.

4. Siemionow MZ, Papay F, Djohan R, et al. First U.S. near-total human face transplantation: a paradigm shift for massive complex injuries. Plast Reconstr Surg 2010: 125: 111-22.

5. Meningaud J-P, Hivelin M, Benjoar M-D, et al. The Procurement of Allotransplants for Ballistic Trauma: A Preclinical Study and a Report of Two Clinical Cases. Plastic and Reconstructive Surgery 2011: 127: 1892-900.

6. Carty MJ, Hivelin M, Dumontier C, et al. Lessons learned from simultaneous face and bilateral hand allotransplantation. Plast Reconstr Surg 2013: 132: 423-32.

7. Diaz-Siso JR, Parker M, Bueno EM, et al. Facial allotransplantation: A 3-year follow-up report. Journal of Plastic, Reconstructive & Aesthetic Surgery 2013: 66: 1458-63.

8. Cavadas PC, Ibáñez J, Thione A. Surgical aspects of a lower face, mandible, and tongue allotransplantation. J Reconstr Microsurg 2012: 28: 43-7.

9. Petruzzo P, Kanitakis J, Testelin S, et al. Clinicopathological Findings of Chronic Rejection in a Face Grafted Patient. Transplantation 2015: 99: 2644-50.

10. Sicilia-Castro D, Gomez-Cia T, Infante-Cossio P, et al. Reconstruction of a severe facial defect by allotransplantation in neurofibromatosis type 1: a case report. Transplant Proc 2011: 43: 2831-7.

11. Barret JP, Serracanta J. LeFort I osteotomy and secondary procedures in full-face transplant patients. J Plast Reconstr Aesthet Surg 2013: 66: 723-5.

12. Lantieri L, Grimbert P, Ortonne N, et al. Face transplant: long-term follow-up and results of a prospective open study. Lancet 2016: 388: 1398-407.

13. Wall A, Bueno E, Pomahac B, Treister N. Intraoral features and considerations in face transplantation. Oral Dis 2016: 22: 93-103.

14. Roche NA, Blondeel PN, Vermeersch HF, et al. Long-Term Multifunctional Outcome and Risks of Face Vascularized Composite Allotransplantation. J Craniofac Surg 2015: 26: 2038-46.

15. Özkan Ö, Özkan Ö, Ubur M, et al. Face allotransplantation for various types of facial disfigurements: A series of five cases. Microsurgery 2018: 38: 834-43.

16. Akdogan N, Ersoy-Evans S, Gokoz O, Erdem Y, Nasir S. Early recognition of chronic rejection in a face allotransplant patient with alopecia. J Cutan Pathol 2021: 48: 1286-97.

17. Özel A, Güçlü ZA, Gülşen A, Özmen S. The Importance of the Condition of the Donor Teeth and Jaws During Allogeneic Face Transplantation. J Craniofac Surg 2015: 26: 1338-41.

18. Dorafshar AH, Bojovic B, Christy MR, et al. Total face, double jaw, and tongue transplantation: an evolutionary concept. Plast Reconstr Surg 2013: 131: 241-51.

19. Bettoni J, Balédent O, Petruzzo P, et al. Role of flow magnetic resonance imaging in the monitoring of facial allotransplantations: preliminary results on graft vasculopathy. Int J Oral Maxillofac Surg 2020: 49: 169-75.

20. Chandraker A, Arscott R, Murphy G, et al. Face Transplantation in a Highly Sensitized Recipient. Military Medicine 2016: 181: 221-26.

21. Maciejewski A, Krakowczyk L, Szymczyk C, et al. The First Immediate Face Transplant in the World. Annals of Surgery 2016: 263: e36-e39.

22. Krakowczyk Ł, Maciejewski A, Szymczyk C, Oleś K, Półtorak S. Face Transplant in an Advanced Neurofibromatosis Type 1 Patient. Ann Transplant 2017: 22: 53-57.

23. Hashem AM, Hoffman GS, Gastman B, et al. Establishing the Feasibility of Face Transplantation in Granulomatosis With Polyangiitis. Am J Transplant 2016: 16: 2213-23.

24. Krezdorn N, Alhefzi M, Perry B, et al. Trismus in Face Transplantation Following Ballistic Trauma. J Craniofac Surg 2018: 29: 843-47.

25. Diep GK, Berman ZP, Alfonso AR, et al. The 2020 Facial Transplantation Update: A 15-Year Compendium. Plast Reconstr Surg Glob Open 2021: 9: e3586.

26. Volokh M, Manturova N, Fisun A, et al. First Russian Experience of Composite Facial Tissue Allotransplantation. Plast Reconstr Surg Glob Open 2019: 7: e2521.

27. Diaz-Siso JR, Sosin M, Plana NM, Rodriguez ED. Face transplantation: Complications, implications, and an update for the oncologic surgeon. J Surg Oncol 2016: 113: 971-5.

28. Norrman A, Nylund K, Ruokonen H, et al. Oral findings and treatment of patients with face transplants in Helsinki. Oral Surgery, Oral Medicine, Oral Pathology and Oral Radiology 2021: 132: 44-51.

29. Razonable RR, Amer H, Mardini S. Application of a New Paradigm for Cytomegalovirus Disease Prevention in Mayo Clinic&#x2019;s First Face Transplant. Mayo Clinic Proceedings 2019: 94: 166-70.

30. Knackstedt R, Siemionow M, Djohan R, et al. Youngest Composite Full-Face Transplant: A Model for Vascularized Composite Allograft in Younger Populations. Annals of Plastic Surgery 2022: 89: 564-72.

31. Kantar RS, Ceradini DJ, Gelb BE, et al. Facial Transplantation for an Irreparable Central and Lower Face Injury: A Modernized Approach to a Classic Challenge. Plast Reconstr Surg 2019: 144: 264e-83e.

32. Lantieri L, Cholley B, Lemogne C, et al. First human facial retransplantation: 30-month follow-up. The Lancet 2020: 396: 1758-65.

33. Govshievich A, Saleh E, Boghossian E, et al. Face Transplant: Current Update and First Canadian Experience. Plast Reconstr Surg 2021: 147: 1177-88.

34. Santanelli di Pompeo F, Longo B, Giovanoli P, et al. Facial Transplantation: Nonimmune-Related Hyperacute Graft Failure-The Role of Perfusion Injury: A Case Report. Ann Plast Surg 2021: 86: 469-75.

35. Kauke M, Panayi AC, Tchiloemba B, et al. Face Transplantation in a Black Patient - Racial Considerations and Early Outcomes. N Engl J Med 2021: 384: 1075-76.

36. Kauke M, Panayi AC, Safi AF, et al. Full facial retransplantation in a female patient-Technical, immunologic, and clinical considerations. Am J Transplant 2021: 21: 3472-80.

37. Ramly EP, Alfonso AR, Berman ZP, et al. The First Successful Combined Full Face and Bilateral Hand Transplant. Plast Reconstr Surg 2022: 150: 414-28.

38. Siemionow M, Papay F, Alam D, et al. Near-total human face transplantation for a severely disfigured patient in the USA. The Lancet 2009: 374: 203-09.

39. Diaz-Siso JR, Parker M, Bueno EM, et al. Facial allotransplantation: a 3-year follow-up report. J Plast Reconstr Aesthet Surg 2013: 66: 1458-63.
